# Polymicrobial Sepsis Chronic Immunoparalysis Is Defined by Diminished Ag-Specific T Cell-Dependent B Cell Responses

**DOI:** 10.3389/fimmu.2018.02532

**Published:** 2018-10-31

**Authors:** Frances V. Sjaastad, Stephanie A. Condotta, Jessica A. Kotov, Kathryn A. Pape, Cody Dail, Derek B. Danahy, Tamara A. Kucaba, Lorraine T. Tygrett, Katherine A. Murphy, Javier Cabrera-Perez, Thomas J. Waldschmidt, Vladimir P. Badovinac, Thomas S. Griffith

**Affiliations:** ^1^Microbiology, Immunology, and Cancer Biology Ph.D. Program, University of Minnesota, Minneapolis, MN, United States; ^2^Department of Pathology, University of Iowa, Iowa City, IA, United States; ^3^Department of Microbiology and Immunology, University of Minnesota, Minneapolis, MN, United States; ^4^Medical Student Summer Research Program in Infection and Immunity, University of Minnesota, Minneapolis, MN, United States; ^5^Interdisciplinary Graduate Program in Immunology, University of Iowa, Iowa City, IA, United States; ^6^Department of Urology, University of Minnesota, Minneapolis, MN, United States; ^7^Medical Scientist Training Program, University of Minnesota, Minneapolis, MN, United States; ^8^Department of Microbiology and Immunology, University of Iowa, Iowa City, IA, United States; ^9^Center for Immunology, University of Minnesota, Minneapolis, MN, United States; ^10^Masonic Cancer Center, University of Minnesota, Minneapolis, MN, United States; ^11^Minneapolis VA Health Care System, Minneapolis, MN, United States

**Keywords:** sepsis, B cells, immune suppression, antibody, CD4 T cells

## Abstract

Immunosuppression is one hallmark of sepsis, decreasing the host response to the primary septic pathogens and/or secondary nosocomial infections. CD4 T cells and B cells are among the array of immune cells that experience reductions in number and function during sepsis. “Help” from follicular helper (Tfh) CD4 T cells to B cells is needed for productive and protective humoral immunity, but there is a paucity of data defining the effect of sepsis on a primary CD4 T cell-dependent B cell response. Using the cecal ligation and puncture (CLP) mouse model of sepsis induction, we observed reduced antibody production in mice challenged with influenza A virus or TNP-KLH in alum early (2 days) and late (30 days) after CLP surgery compared to mice subjected to sham surgery. To better understand how these CD4 T cell-dependent B cell responses were altered by a septic event, we immunized mice with a Complete Freund's Adjuvant emulsion containing the MHC II-restricted peptide 2W1S_56−68_ coupled to the fluorochrome phycoerythrin (PE). Immunization with 2W1S-PE/CFA results in T cell-dependent B cell activation, giving us the ability to track defined populations of antigen-specific CD4 T cells and B cells responding to the same immunogen in the same mouse. Compared to sham mice, differentiation and class switching in PE-specific B cells were blunted in mice subjected to CLP surgery. Similarly, mice subjected to CLP had reduced expansion of 2W1S-specific T cells and Tfh differentiation after immunization. Our data suggest CLP-induced sepsis impacts humoral immunity by affecting the number and function of both antigen-specific B cells and CD4 Tfh cells, further defining the period of chronic immunoparalysis after sepsis induction.

## Introduction

Vaccination or infection is one of the most effective ways to generate immunity to microbes. Efficacious vaccinations and natural infection elicit antibody (Ab) production by B cells and their progeny, providing a first line of defense against subsequent microbial invasion. B cells recognize a wide variety of antigens (Ag), including proteins, lipids, polysaccharides, nucleic acids, and chemicals that bind to surface IgM or IgD (1). While serving as a major means of protection against extracellular pathogens and the various toxins they produce, Ab are also a vital means of defense against intracellular pathogens (including viruses) because of their ability to neutralize the pathogen before they can enter a cell, preventing the spread of infection (2, 3). Ab responses can be classified as “T cell-dependent or –independent,” based on the use of CD4 T cell help (4). B cell responses to protein Ag in the absence of CD4 T cell help are weak, producing Ab with low affinity. In contrast, B cell responses generated with the help of CD4 T cells produce high affinity, class-switched Ab.

There has been considerable interest in recent years in CXCR5^+^PD-1^+^Bcl6^+^ follicular helper CD4 T (Tfh) cells—the specialized CD4 T cell subset that provides help to B cells—and understanding the role they play in facilitating the proliferation and function of primary and memory B cells (5, 6). When Tfh cells detect B cells presenting their cognate Ag, they upregulate CD154 expression and secrete a number of cytokines to promote B cell proliferation and differentiation into plasma cells (7, 8). During the early Ab response plasma cells secrete Ab and some degree of isotype switching occurs. A few of the activated B cells return to the follicle, accompanied by Tfh cells, where they proliferate and form a germinal center (GC) in response to the Tfh cell-derived signals. The proliferating GC B cells undergo immunoglobulin (Ig) heavy chain isotype switching, somatic hypermutation of Ab gene variable regions, and affinity maturation. Repeated exposure to their cognate Ag promotes the B cells to produce the highest affinity and most efficacious Ab for neutralization of microbes and their toxic products and differentiate into long-lived plasma cells and memory B cells (9–11).

The importance of both the humoral and cellular arms of the adaptive immune system for overall health is dramatically illustrated by individuals with immune system defects being highly susceptible to serious and often life-threatening infections. States of immune deficiency can be congenital (e.g., impaired T and/or B cell development) or acquired (e.g., HIV infection, iatrogenic (post-organ transplant) immune suppression, or surgery/trauma). The combination of quantitative and qualitative impairments to multiple compartments of the immune system that develop in the wake of a septic event lead to an acquired immune deficiency (12). Sepsis, currently defined as life-threatening organ dysfunction resulting from the dysregulated host response to infection (13), is responsible for thousands of deaths annually (14). As the host recovers from the initial septic event, the immune system becomes hyporesponsive, resulting in a long-lasting immunosuppressive state. Advances in critical care and life support medicine have greatly improved survival rates of patients in the initial hyperinflammatory phase of sepsis, such that the acute cytokine storm is responsible for only ~30% of the sepsis-related mortality. Today the majority of sepsis-related deaths occur after the patient has recovered from the initial hyperinflammatory phase, with many patient deaths occurring weeks and months later (15, 16). Reduced numbers of immune cells in septic patients contribute to the decreased responses to new and secondary infections (17, 18). While the characteristic sepsis-induced lymphopenia is transient, the prolonged immune suppression that develops after a septic event and remains even once lymphocyte numbers normalize is now considered a leading cause of prolonged susceptibility to secondary pathogens normally handled by the immune system in healthy individuals (19).

Studies in human septic patients show both CD4 T cells and B cells are reduced during the hyperinflammatory phase of sepsis (20), but there is limited data detailing the long-term impact of sepsis on these cells within the context of a CD4 T cell-dependent B cell response. We have taken advantage of using peptide:MHC I or II tetramers to track the number and function of endogenous Ag-specific CD8 or CD4 T cell populations (21–23) to investigate how specific subsets of the T cell compartment are quantitatively and qualitatively affected in the mouse model of cecal ligation and puncture (CLP)-induced polymicrobial sepsis (24). Similar approaches can identify endogenous Ag-specific B cells, such as B cells specific for the commonly used fluorochrome phycoerythrin (PE) (25–27). The objective of this study was to define the mechanism(s) responsible for the impairment of primary CD4 T cell-dependent B cell responses in the septic host using the CLP model followed by immunization with an Ag containing defined CD4 T cell and B cell epitopes. Our data suggest CLP-induced sepsis impacts humoral immunity by affecting the number and function of both Ag-specific B cells and CD4 Tfh cells.

## Materials and methods

### Mice

8 week-old female C57BL/6 mice were purchased from the National Cancer Institute (Frederick, MD) and maintained in AALAC-approved animal facilities at the University of Minnesota and University of Iowa at the appropriate biosafety level. Experimental procedures were approved by the University of Minnesota and University of Iowa Institutional Animal Care and Use Committees and performed following the Office of Laboratory Animal Welfare guidelines and PHS Policy on Human Cancer and Use of Laboratory Animals.

### Cecal ligation and puncture (CLP)

Sepsis was induced by CLP (24). Briefly, mice were anesthetized using isoflurane (2.5% gas via inhalation) or Ketamine/xylazine (87.5 and 12.5 mg/kg, respectively, i.p.). The abdomen was shaved and disinfected with 5% povidone-iodine antiseptic. Bupivicaine (6 mg/kg s.c.) was then administered at the site where a midline incision was made. The distal third of the cecum was ligated with 4-0 silk suture and punctured once with a 25-g needle to extrude a small amount of cecal content. The cecum was returned to the abdomen, the peritoneum was closed via continuous suture, and the skin was sealed using surgical glue (Vetbond; 3M, St. Paul, MN). Meloxicam (2 mg/kg) in 1 ml saline was administered at the conclusion of surgery and the following 3 days for post-operative analgesia and fluid resuscitation. Mice were monitored daily for weight loss and pain for at least 5 days post-surgery. To control for non-specific changes from the surgery, sham mice underwent the same laparotomy procedure excluding ligation and puncture.

### Immunizations

On days 2 or 30 after sham or CLP surgery, B6 mice were immunized with the following reagents: (1) influenza A virus (A/PR/8; 10^5^ PFU in 100 μl PBS i.p.; obtained from Dr. Ryan Langlois, University of Minnesota); (2) 2,4,6 trinitrophenyl-conjugated keyhole limpet hemocyanin [TNP-KLH; 50 μg i.p. (Biosearch Technologies, Novato, CA)] precipitated in alum (100 μg) or mixed with CpG containing oligonucleotide 1826 (10 μg; TCCATGA*CG*TTCCTGA*CG*TT), followed 3 weeks later by a second immunization; or (3) 2W1S:PE conjugates [i.p. injection of 0.6 μg 2W1S peptide (EAWGALANWAVDSA; GenScript, Piscataway, NJ) conjugated to 2.4 μg PE (ProZyme; Hayward, CA) emulsified in Complete Freund's Adjuvant (CFA; Sigma-Aldrich, St. Louis, MO)] (28). The 2W1S:PE conjugate was formed by combining biotinylated 2W1S peptide with streptavidin-PE at a 4:1 ratio.

### Enrichment and analysis of Ag-specific B cells and CD4 T cells and B cells

To quantify the number of PE-specific B cells and 2W1S-specific CD4 T cells in mice following sham or CLP surgery, an enrichment protocol was used (25–27, 29). Briefly, spleens and peripheral LN (axillary, brachial, cervical, inguinal, and mesenteric) were harvested for each mouse analyzed. Pooled LN [in 1 ml of FACS buffer (PBS containing 0.1% NaN_3_ and 2% FBS)] were mashed on a nylon mesh into a single-cell suspension. The spleen from the same mouse was then added, along with 1 ml of RPMI-1640 medium containing Collagenase P (0.2 mg/ml final), Dispase (0.8 mg/ml final), and DNase I (01 mg/ml final). A single-cell suspension from these pooled lymphoid tissues was then generated using a GentleMACS dissociator (Miltenyi Biotech). This suspension was incubated in a 37°C water bath for 20 min, and then run on the GentleMACS a second time. Ten (10) ml of ice cold FACS buffer containing 5 mM EDTA was added to the dissociator tubes, which were inverted several times to wash the top of the tubes before decanting into new 50 ml conical tubes. The dissociator tubes were rinsed with an additional 5 ml of ice cold FACS buffer, which was then decanted into the corresponding 50 ml conical tubes. The cells were pelleted by centrifugation, and then resuspended in 400 μl FACS buffer containing 5 mM EDTA and anti-CD16/32 mAb (clone 93, 1:100 dilution; BioLegend) to block Fc receptors. In some cases, the single-cell suspension was divided to permit separate enrichments for the B cells and CD4 T cells from the same sample.

#### B cell enrichment

Cells were incubated with PE (1 μg; Prozyme, Hayward, CA) for 30 min on ice. After washing with 10 ml cold FACS buffer with 5 mM EDTA, the cells were then incubated with 25 μl anti-PE-conjugated magnetic microbeads (Miltenyi Biotec) for 30 min on ice. The cells were washed, resuspended in 3 ml FACS buffer, and then passed over a magnetized LS column to enrich for the PE-specific cells. The column was washed twice with 3 ml of FACS buffer, and the bound cells were eluted from the column by pushing 5 ml of buffer with a plunger.

#### CD4 T cell enrichment

I-A^b^-specific tetramers containing 2W1S (EAWGALANWAVDSA) were used to identify and enrich Ag-specific CD4 T cells (29–31). Briefly, biotinylated I-A^b^ molecules containing the 2W1S peptide covalently linked to the I-A^b^ β chain were produced in *Drosophila melanogaster* S2 cell along with the I-A^b^ α chain (29). The monomers were purified, and then made into tetramers with streptavidin-allophycocyanin (SA-APC; Prozyme). Tetramers (10 nM final concentration) were then added to single-cell suspensions in 300 μl tetramer staining buffer (PBS containing 5% FBS, 2 mM EDTA, and 50 μ? Dasatinib, 1:50 normal mouse serum, and 1:100 anti-CD16/32 mAb). The cells were incubated in the dark at room temperature for 1 h, followed by a wash in 10 ml ice cold FACS Buffer. The tetramer-stained cells were then resuspended in 300 μl FACS Buffer, mixed with 25 μl of anti-APC mAb-conjugated magnetic microbeads (StemCell Technologies), and incubated in the dark on ice for 30 min. The cells were washed, resuspended in 3 ml cold FACS Buffer, and passed through an EasySep Magnet (StemCell Technologies) to yield an enriched tetramer positive population.

The resulting enriched fractions were stained with a cocktail of fluorochrome-labeled mAb (see below). Cell numbers for each sample were determined using AccuCheck Counting Beads (Invitrogen). Samples were then analyzed using an LSR II flow cytometer (BD) and FlowJo software (TreeStar Inc., Ashland, OR). The percentage of PE^+^ or 2W1S:I-A^b+^ events was multiplied by the total number of cells in the enriched fraction to calculate the total number of PE-specific B cells or 2W1S:I-A^b^-specific CD4 T cells, respectively.

### Flow cytometry

To assess the expression of cell surface proteins, cells were incubated with fluorochrome-conjugated mAb at 4°C for 30 min. The cells were then washed with FACS buffer. For some experiments, the cells were then fixed with PBS containing 2% paraformaldeyhe. In procedures requiring intracellular staining, cells were permeabilized following surface staining using the transcription factor staining kit (eBioscience), stained for 1 h at 4°C with a second set of fluorochrome-conjugated mAb, and suspended in FACS buffer for acquisition. The fluorochrome-conjugated mAb used in surface and intracellular staining were as follows: *B cell panel*—FITC IgA, PerCP-eF710 IgM, AF594 IgG3, AF647 IgG2b, AF700 CD38, APC-eF780 “dump” (CD90.2, CD11c, F4-80, and GR1), BV510 IgE, BV605 IgG1, BV711 IgG2A, BV786 IgD, AF350 IgG (H+L), BUV395 B220; *T cell surface panel* –PE-Cy7 PD-1, AlexaFluor® (AF) 700 CD44, APC-eFluor® (eF) 780 “dump” (CD11b, CD11c, and B220), Brilliant Violet™ (BV) 421 CXCR5, BV650 CD8a, and Brilliant Ultraviolet™ (BUV) 395 CD4; and *T cell ICS panel*—AF488 Bcl6, BV605 Tbet, AF700 CD44, APC-eF780 “dump” (CD11b, CD11c, and B220), and BUV395 CD4.

### Assessment of Ab production after TNP-KLH or influenza a virus immunization

Mice immunized with TNP-KLH were bled 7 days after the boost to collect serum to measure TNP-specific Ab levels. Mice challenged with influenza A virus were bled after 28 days to collect serum to measure anti-IAV Ab titers. Mice were anesthetized and blood was collected retro-orbitally. Blood samples were clotted and separated serum was stored at −80°C until use in enzyme-linked immunosorbent assay (ELISA) to determine the presence of Ag-specific Ab.

TNP-specific Ab were determined as follows: 96-well ELISA plates (Immulon 2, Thermo, Milford, MA) were coated with goat anti-mouse IgM (10 μg/ml; Southern Biotech, Birmingham, AL), goat anti-mouse IgG1 (5 μg/ml; Southern Biotech), or goat anti-mouse IgG2b (5 μg/ml; Southern Biotech) in 0.05 M Tris-HCl buffer (pH 9.5) overnight at 4°C. Coated plates were blocked with 5% w/v dry milk in phosphate buffered saline (PBS). Control anti-TNP mAb (for standard curves) or serum samples appropriately diluted in 5% dry milk-PBS were added, and similarly incubated. After washing, 2μg/ml TNP-human gamma globulin-biotin diluted in 5% dry milk-PBS was added to each well, and the plates further incubated. Alkaline phosphatase streptavidin (3 μg/ml; Zymed, San Francisco, CA) diluted in 5% dry milk-PBS was added after washing. Substrate (2 mg/ml; Sigma Chemical Co., St. Louis, MO) diluted in substrate buffer [50 mM Na_2_CO_3_ and 1 mM ${\text{MgCl}}_{2}^{.}$ 6H_2_O in H_2_O (pH 9.8)] was added to each well, and absorbance measured at a dual wavelength of 405 and 540 nm using a Microplate Autoreader EL311 (Bio-Tek Instruments, Winooski, VT). All washes between steps were performed with a 0.9% NaCl, 0.05% Tween-20 buffer (pH 7.0) and all incubation steps were done at 37°C in 5% CO_2_. Ab concentrations were determined from standard curves using DeltaSOFT software (Bio-Tek Instruments). Control mAb used for standard curves were 49.2 (mouse IgG2b anti-TNP mAb; Pharmingen, San Diego, CA), 4G2F8 (mouse IgM anti-TNP mAb), and 1B7 (mouse IgG1 anti-TNP mAb. 4G2F8 and 1B7 were affinity purified by passage of hybridoma culture supernatants over TNP-bovine gamma globulin-Sepharose 6B followed by elution with TNP-glycine (Sigma Chemical Co.).

Influenza-specific Ab were determined as follows: 96-well ELISA plates were coated with purified A/PR/8 Influenza A virus (50 μl/well of 2 mg/ml PBS virus) overnight at 4°C. Coated plates were blocked for 1 h at room temperature with 5% normal goat or donkey serum in PBS, followed by incubation with diluted serum samples from IAV-challenged mice overnight at 4°C. After washing, plates were incubated with either an alkaline phosphatase-conjugated goat anti-mouse Ig (Southern Biotech) or donkey anti-mouse IgG (Jackson ImmunoResearch). Substrate was added and absorbance was measured as described above.

### Statistical analyses

Data shown are presented as mean values ± SEM. GraphPad Prism 7 was used for statistical analysis, where statistical significance was determined using two-tailed Student *t*-test (for 2 individual groups, if unequal variance Mann-Whitney U test was used) or group-wise, one-way ANOVA analyses followed by multiple-testing correction using the Holm-Sidak method, with α = 0.05. ^*^p < 0.05, ^**^p < 0.01, ^***^p < 0.005, ^****^p < 0.001.

## Results

### Sepsis induces a transient reduction in B cells and CD4 T cells

Patients surviving a septic event often have suppressed immune function, as data showing reduced immune cell function in critically ill sepsis patients date back over 40 years (32). While some data suggested a phenotypic switch in CD4 T cells from Th1 to Th2 (33), other data indicated that the reduced cellular activity was more likely due to a global dysfunction (34). This idea is reinforced by decreased expression of Tbet, GATA3, and RORγt, the transcription factors regulating Th1, Th2, and Th17 phenotypes, respectively, in CD4 T cells from septic patients (35). More recently, post-mortem assessment of T cells from patients who died from severe sepsis showed almost no production of IFNγ, TNFα, IL-6, and IL-10 after anti-CD3/CD28 mAb stimulation compared to samples from non-septic, control patients (36)—further supporting the idea that sepsis affects general T cell function. Indirect evidence of defective CD4 T cell function has come from other studies describing altered humoral responses after sepsis, specifically in terms of Ag-specific immunity (e.g., T cell-dependent Ab responses) (37, 38). With this clinical information in mind, we wanted to further investigate how sepsis affects the generation of a primary CD4 T cell-dependent B cell response using the CLP mouse model of polymicrobial sepsis. The severity of the CLP we performed was marked by a significant, but transient, loss of weight that was recovered by 7 days after surgery (Supplemental Figure 1A), as well as the rapid production of IL-1β, IL-6, IFNγ, and TNF detectable in the serum during the first 24 h after surgery (Supplemental Figure 1B). Both of these parameters are consistent with previous reports (39–43). In addition, we see a mortality rate of ~25% in the group of mice receiving CLP surgery (Supplemental Figure 1C), which is consistent with clinical rates (44).

**Figure 1:**
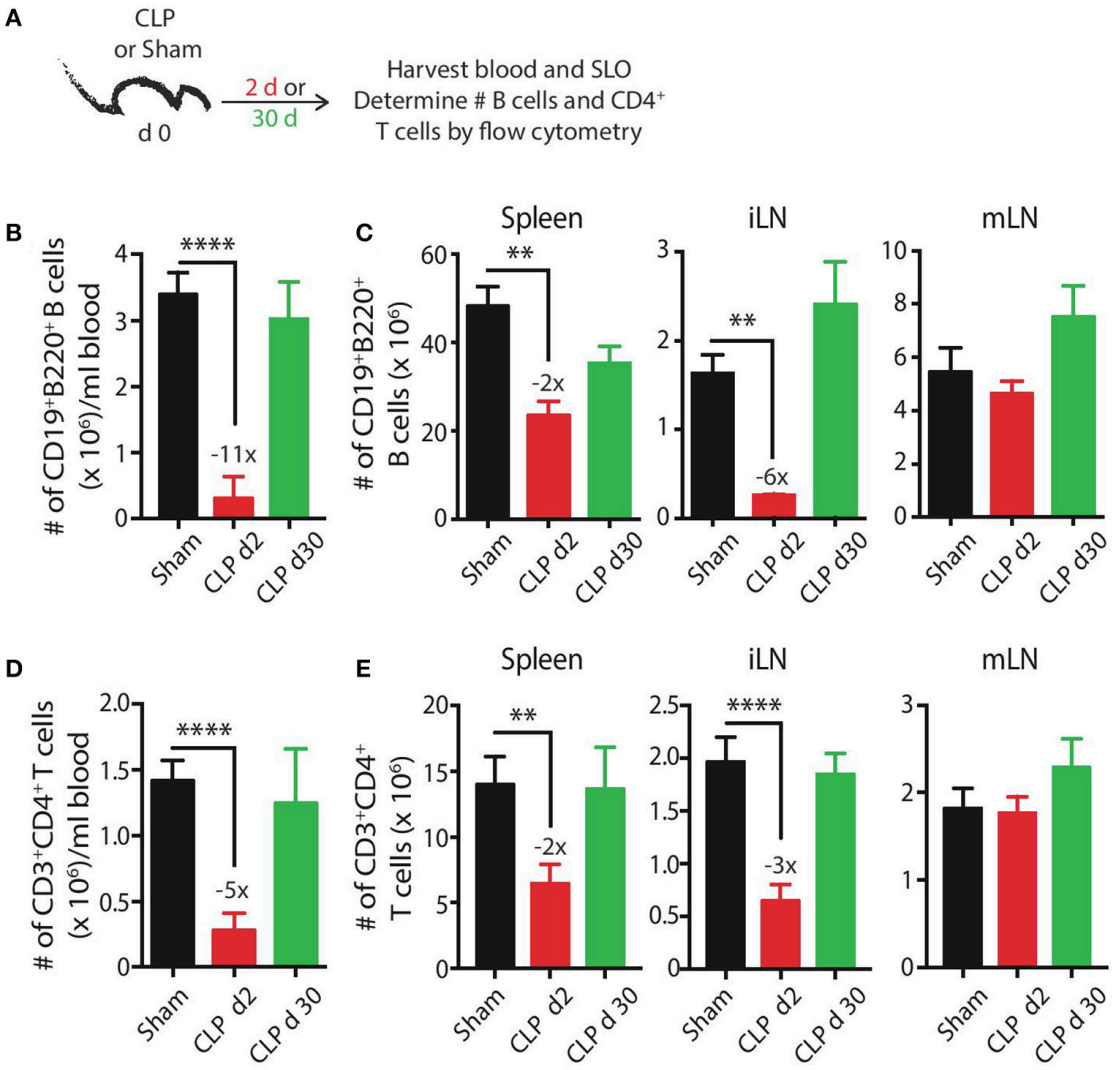
CLP induces transient reductions in the number of B cells and CD4 T cells**. (A)** Experimental design—blood and secondary lymphoid organs (SLO) including spleens, inguinal lymph nodes (iLN), and mesenteric lymph nodes (mLN) were harvested 2 and 30 days after sham or CLP surgery. The number of **(B,C)** CD19^+^ B220^+^ B cells or **(D,E)** CD3^+^ CD4^+^ T cells in blood (per ml), spleen, iLN, and mLN was determined by flow cytometry. *n* = 5–7 mice/group. **p < 0.01, ****p < 0.001. Numbers above bars indicate the average fold change in number compared to sham mice. Data are representative of at least 3 independent experiments.

We initially wanted to define the numerical changes that occur within the total B cell and CD4 T cell compartments—the cells that participate in CD4 T cell-dependent B cell responses—following a septic event. B cells and CD4 T cells present in the blood and secondary lymphoid organs were enumerated by flow cytometry early (day 2) and late (day 30) after sham or CLP surgery (Figure 1A). B cell numbers in the blood, spleen, and inguinal lymph nodes (iLN) were significantly reduced 2 days after sepsis induction, decreasing 11-fold, 2-fold, and 6-fold, respectively, before recovering to sham levels by day 30 (Figures 1B,C). Interestingly, there was no reduction in B cells isolated from mesenteric lymph nodes (mLN) on day 2 and there was a slight (but insignificant) increase in number on day 30. Similar trends were observed with CD4 T cells—transient numerical reductions in the blood (5-fold), spleens (2-fold), and iLN (3-fold) but no change in the mLN (Figures 1D,E). The mLN drain the gut mucosa and are located within the site of the initial polymicrobial septic insult, suggesting the proximity to the intraperitoneal inflammation may either prevent the sepsis-induced death of lymphocytes seen in the periphery and/or recruit cells from periphery through the production of inflammatory cues. However, migration of cells to mLN cannot fully account for the diminished cellularity observed in other tissues. We have previously shown dendritic cells follow the same pattern of numerical reduction, with losses in the blood, spleen, and iLN, and no change in the mLN (45), indicating cells of the lymphoid and myeloid lineages are similarly maintained numerically in the anatomical locations where the nidus of the septic event is found. Data examining the B cell and CD4 T cell compartments at the “total population” level suggest the immune system has returned to its pre-sepsis state by day 30 in terms of B cell and CD4 T cell numbers.

### Sepsis-induced effects on Ag-specific B cells and CD4 T cell

The results presented in Figure 1 suggest the re-establishment of a “normal” immune system within 30 days after CLP-induced sepsis. However, previous work from our group revealed distinct differences in the ability of individual Ag-specific CD4 T cell populations to numerically and functionally recover after sepsis (22). We hypothesized that similar differences may occur for an individual Ag-specific B cell population within the total B cell compartment, prompting us to employ a system where we could directly monitor defined populations of Ag-specific B cells and CD4 T cells within the total B cell and CD4 T cell compartments to more rigorously study the effect of sepsis on CD4 T cell-dependent B cell responses. Specifically, we used enrichment protocols to identify B cells specific for the fluorchrome phycoerythrin (PE) (25–27) and CD4 T cells specific for the 2W1S variant of peptide 52-68 from the I-E α-chain (29, 46).

**Figure 2:**
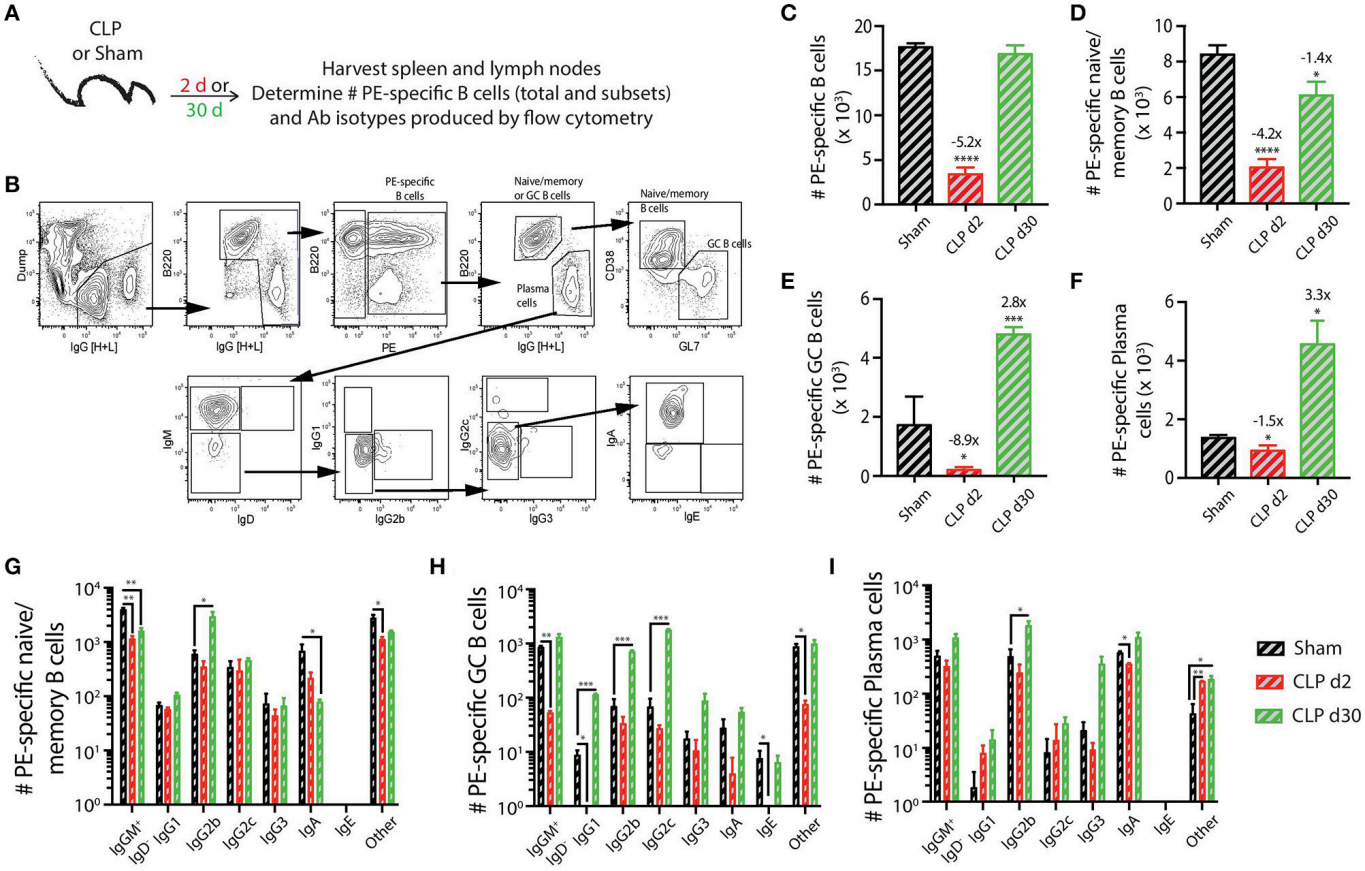
Numerical changes in PE-specific B cells after sepsis. **(A)** Experimental design—Spleens and peripheral lymph nodes (axillary, brachial, cervical, inguinal, and mesenteric) were harvested from mice on day 2 and 30 post-sham or CLP surgery and combined. **(B)** Gating scheme used to identify PE-specific B cells, as well as the B220^hi^IgG [H+L]^lo^ CD38^+^GL7^−^ naïve/memory B cells, B220^hi^IgG [H+L]^lo^ CD38^−^GL7^+^ germinal center (GC) B cells, and B220^lo^IgG [H+L]^hi^CD38^−^GL7^−^ plasma cells. This representative scheme also shows the gating used to determine the extent of class switching within the plasma cell population, but similar gating was used on the naïve/memory and GC B cell populations. Representative flow plots are from a mouse that underwent sham surgery. The number of **(C)** total PE-specific B cells and PE-specific **(D)** naïve/memory B cells, defined as B220^hi^ IgG [H+L]^lo^ CD38^+^GL7^−^, **(E)** germinal center (GC) B cells, defined as B220^hi^ IgG [H+L]^lo^ CD38^−^ GL7^+^, and **(F)** plasma cells, defined as B220^lo^ IgG [H+L]^hi^ CD38^−^ GL7^−^, were determined. Numbers above bars indicate the average fold change in number compared to sham mice. **(G–I)** The naïve/memory B cell, GC B cell, and plasma cell subsets from the unimmunized mice were additionally subdivided based on the Ab isotype being produced. *n* = 5–7 mice/group. Statistical comparisons were made between sham mice and either CLP d2 or d30 mice, where *p < 0.05, **p < 0.01, ****p < 0.001. Data are representative of at least 3 independent experiments.

As a first step in this analysis, we determined how sepsis affected the number and phenotype of PE-specific B cells (Figure 2A). We were able to clearly detect and quantify the PE-specific B cells, as well as the B220^hi^IgG [H+L]^int^CD38^+^GL7^−^ naïve/memory B cells, B220^hi^IgG [H+L]^int^CD38^−^GL7^+^ germinal center (GC) B cells, and B220^lo^IgG [H+L]^hi^CD38^−^GL7^−^ plasma cells within the PE-specific B cell population (Figure 2B). Similar to the transient loss and recovery within the total B cell compartment (Figures 1B,C), there was a significant reduction (5.2-fold less compared to sham mice) in number of PE-specific B cells 2 days after CLP surgery that recovered by day 30 (Figure 2C). The number of PE-specific naïve/memory B cells decreased ~4-fold 2 days after CLP surgery, but did not fully recover by day 30 to the number found in mice that underwent sham surgery (Figure 2D). Interestingly, while there were ~9- and 1.5-fold reductions in number of PE-specific GC B cells and plasma cells, respectively, 2 days after CLP surgery, this was followed by a ~3-fold increase in both of these subsets by day 30 after CLP surgery (Figures 2E,F). In addition to determining the number of these PE-specific B cell subsets, we also evaluated how the septic event affected the Ab isotypes they produced. As expected, there were numerical reductions in several populations 2 days after CLP surgery, including IgM^+^IgD^−^ naïve/memory, IgG1^+^ and IgE^+^ GC B cells, and IgA^+^ plasma cells (Figures 2G–I). It is also important to note several populations were increased by day 30 after CLP, namely the IgG2b-producing PE-specific naïve/memory B cells, GC B cells, and plasma cells 30 days after CLP compared to sham mice (Figures 2G–I). IgG1- and IgG2c-producing GC B cells also increased in number by day 30 after CLP surgery. These data show sepsis can induce a variety of small numerical changes within the B cell compartment when examined at the Ag-specific level.

**Figure 3:**
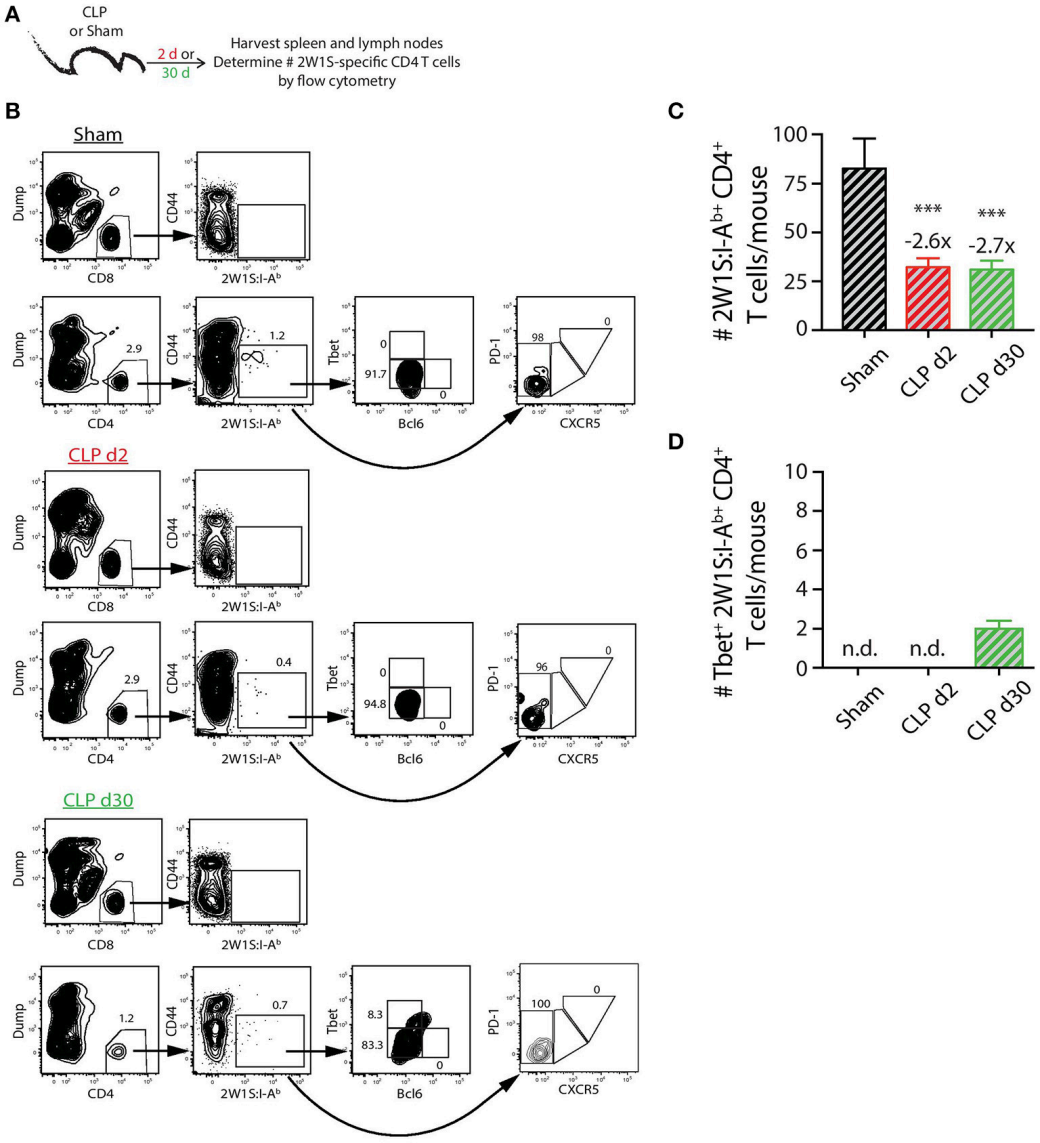
Numerical changes in 2W1S-specific CD4 T cells after sepsis. **(A)** Experimental design—Spleens and peripheral lymph nodes (axillary, brachial, cervical, inguinal, and mesenteric) were harvested from mice on day 2 and 30 post-sham or CLP surgery and combined. **(B)** Gating scheme used to identify 2W1S:I-A^b^-specific CD4 T cells (CD8 T cells were used as the internal negative control for tetramer binding), as well as the 2W1S:I-A^b^-specific CD4 T cells expressing Tbet, Bcl-6, or PD-1 and CXCR5. The frequency of cells within the gated populations is indicated. The number of **(C)** total and **(D)** Tbet^+^ 2W1S:I-A^b^-specific CD4 T cells was determined. *n* = 9–10 mice/group in **(B,C)**. Statistical comparisons were made between sham mice and either CLP d2 or d30 mice, where ***p < 0.005. Numbers above bars indicate the average fold change in number compared to sham-treated mice.

In contrast to the PE-specific B cells and consistent with our previous data (22), we did not observe the same numerical recovery of 2W1S:I-A^b^-specific T cells after CLP surgery that was seen for the total CD4 T cell population. In fact, the number of 2W1S:I-A^b^-specific CD4 T cells was reduced ~3-fold at day 2 post-CLP, which was maintained at day 30 (Figures 3A–C). Our analysis of the 2W1S:I-A^b^-specific T cells also included an assessment of CD4 T cell lineage-specific master regulators Tbet (Th1) and Bcl6 (Tfh) (47, 48). A small number of the 2W1S:I-A^b^-specific CD4 T cells expressed Tbet by day 30 after CLP surgery (Figure 3D), but we were unable to detect any 2W1S:I-A^b^-specific CD4 T cells expressing Bcl6 or PD-1 and CXCR5, indicators of Tfh differentiation, after CLP. Thus, the data in Figures 2, 3 show how sepsis affects the number of PE-specific B cells and 2W1S:I-A^b^-specific CD4 T cells.

### Prolonged impairment in primary CD4 T cell-dependent B cell responses is associated with reduced germinal center T follicular helper (Tfh) cell differentiation

**Figure 4:**
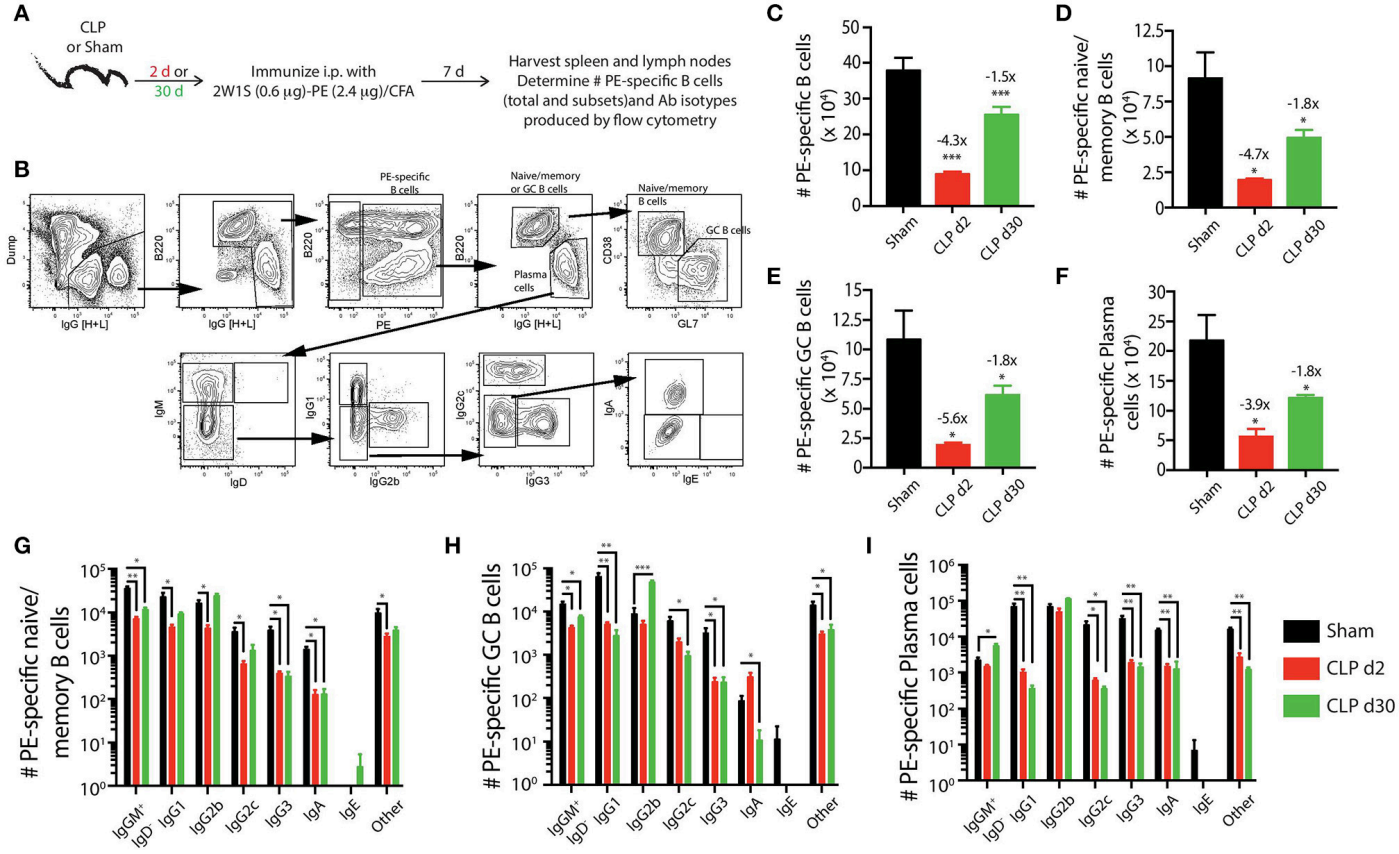
Sepsis hinders B cell differentiation and class-switching during a CD4 T cell-dependent B cell response. **(A)** Experimental design—mice underwent sham or CLP surgery and were then immunized on day 2 or 30 after surgery with 2W1S-PE/CFA i.p. Spleens and peripheral lymph nodes were harvested 7 days after immunization and combined. **(B)** Gating scheme used to identify PE-specific B cells, as well as the B220^hi^IgG [H+L]^lo^ CD38^+^GL7^−^ naïve/memory B cells, B220^hi^IgG [H+L]^lo^ CD38^−^GL7^+^ germinal center (GC) B cells, and B220^lo^IgG [H+L]^hi^CD38^−^GL7^−^ plasma cells. This representative scheme also shows the gating used to determine the extent of class switching within the plasma cell population, but similar gating was used on the naïve/memory and GC B cell populations. Representative flow plots are from a mouse immunized 2 days after sham surgery. The number of **(C)** total PE-specific B cells and PE-specific **(D)** naïve/memory B cells, **(E)** GC B cells, and **(F)** plasma cells were determined. Numbers above bars indicate the average fold change in number compared to sham-treated mice. **(G–I)** The naïve/memory B cell, GC B cell, and plasma cell subsets from the immunized mice were additionally subdivided based on the Ab isotype being produced. *n* = 5–7 mice/group. Statistical comparisons were made between sham mice and either CLP d2 or d30 mice, where *p < 0.05, **p < 0.01, and ***p < 0.005. Data are representative of at least 3 independent experiments.

We next determined how sepsis affected the ability of the PE-specific B cells and 2W1S:I-A^b^-specific CD4 T cells to respond after immunization with a CFA emulsion containing the MHC II-restricted 2W1S_56−68_ peptide coupled to PE (28) (Figure 4A). The 2W1S-PE immunogen is internalized by the B cell receptor of PE-specific B cells, which then present 2W1S in MHC II complexes to 2W1S-specific CD4 T cells who provide the necessary help to generate a robust B cell response. This immunization method allowed us to simultaneously track the numerical and phenotypic changes among PE-specific B cells and 2W1S:I-A^b^-specific CD4 T cells in the same mouse during a CD4 T cell-dependent B cell response.

Using the identical Ab panels and gating schemes that were used above (Figure 4B) to identify PE-specific B cells in mice that had only experienced sham or CLP surgery, we found the immunization-induced expansion for each PE-specific B cell population was greatest in the sham-treated mice (Figures 4C–F). While the number of total PE-specific B cells and each subset had recovered or expanded by day 30 after CLP surgery, the response of these populations to immunization with 2W1S-PE/CFA was significantly reduced in mice subjected to CLP surgery compared to sham mice. We noted 4–6-fold fewer PE-specific naïve/memory, GC, and plasma cells in mice immunized 2 days after CLP surgery compared to sham mice, and this numerical reduction (~2-fold less) was maintained in the CLP mice immunized 30 days after surgery. Thus, the PE-specific B cell compartment showed long-lasting functional impairment in terms of cellular proliferative capacity, extending well-past the resolution of the septic event. Sepsis also affected the degree of class switching after immunization with 2W1S-PE/CFA for each subset of PE-specific B cells. In general, there were reductions in the number of multiple isotypes produced by the PE-specific B cell subsets (Figures 4G–I). This was most evident in the PE-specific plasma cells, as there were significantly fewer cells producing IgG1, IgG2c, IgG3, and IgA after immunization either on day 2 or 30 after CLP surgery (Figure 4I).

**Figure 5:**
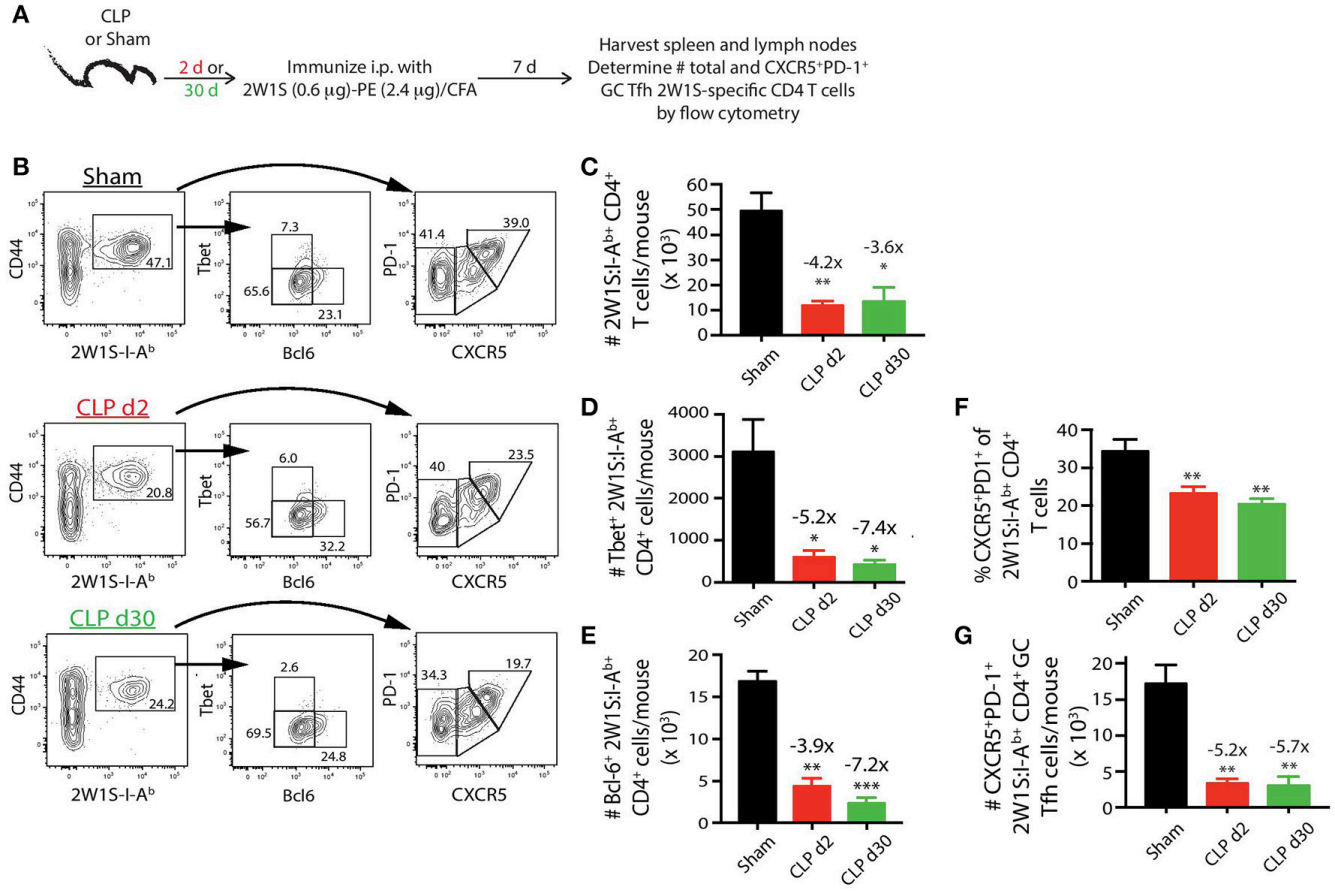
Sepsis restricts the ability of 2W1S:I-A^b^-specific CD4 T cells to differentiate into Tfh during a CD4 T cell-dependent B cell response. **(A)** Experimental design—mice underwent sham or CLP surgery and were then immunized on day 2 or 30 after surgery with 2W1S-PE/CFA i.p. Spleens and peripheral lymph nodes were harvested 7 days after immunization and combined. **(B)** Gating scheme used to identify 2W1S:I-A^b^-specific CD4 T cells (CD8 T cells were used as the internal negative control for tetramer binding), as well as the 2W1S:I-A^b^-specific CD4 T cells expressing Tbet, Bcl-6, or PD-1 and CXCR5. The frequency of cells within the gated populations is indicated. The number of **(C)** total, **(D)** Tbet^+^, **(E)** Bcl-6^+^ 2W1S:I-A^b^-specific CD4 T cells, as well as **(F)** frequency, and **(G)** number of 2W1S:I-A^b^-specific CD4 T cells expressing CXCR5 and PD-1, was determined. *n* = 5–7 mice/group. Statistical comparisons were made between sham-treated mice and either CLP d2- or d30-treated mice, where *p < 0.05, **p < 0.01, and ***p < 0.005. Data are representative of at least 3 independent experiments.

The extent of Tfh cell differentiation by the 2W1S:I-A^b^-specific CD4 T cells after 2W1S-PE/CFA immunization (Figure 5A) was also impaired by sepsis. There were ~4-fold fewer total 2W1S:I-A^b^-specific CD4 T cells in the mice subjected to CLP surgery, regardless of when the immunization occurred (day 2 or 30 after surgery), compared to sham mice (Figures 5B,C). Moreover, there was a significant reduction in the frequency and number (~5-fold less than sham-treated mice) of 2W1S:I-A^b^-specific CD4 T cells that differentiated into GC Tfh cells, based on expression of Bcl-6 (Figure 5E) or CXCR5 and PD-1 (Figures 5F,G). The data in Figures 2–5 reveal the profound quantitative and qualitative effects of sepsis on both Ag-specific B cells and CD4 T cells, especially after immunization with a model Ag designed to elicit a CD4 T cell-dependent B cell response, that were not apparent when evaluating the bulk B cell and CD4 T cell compartments.

**Figure 6:**
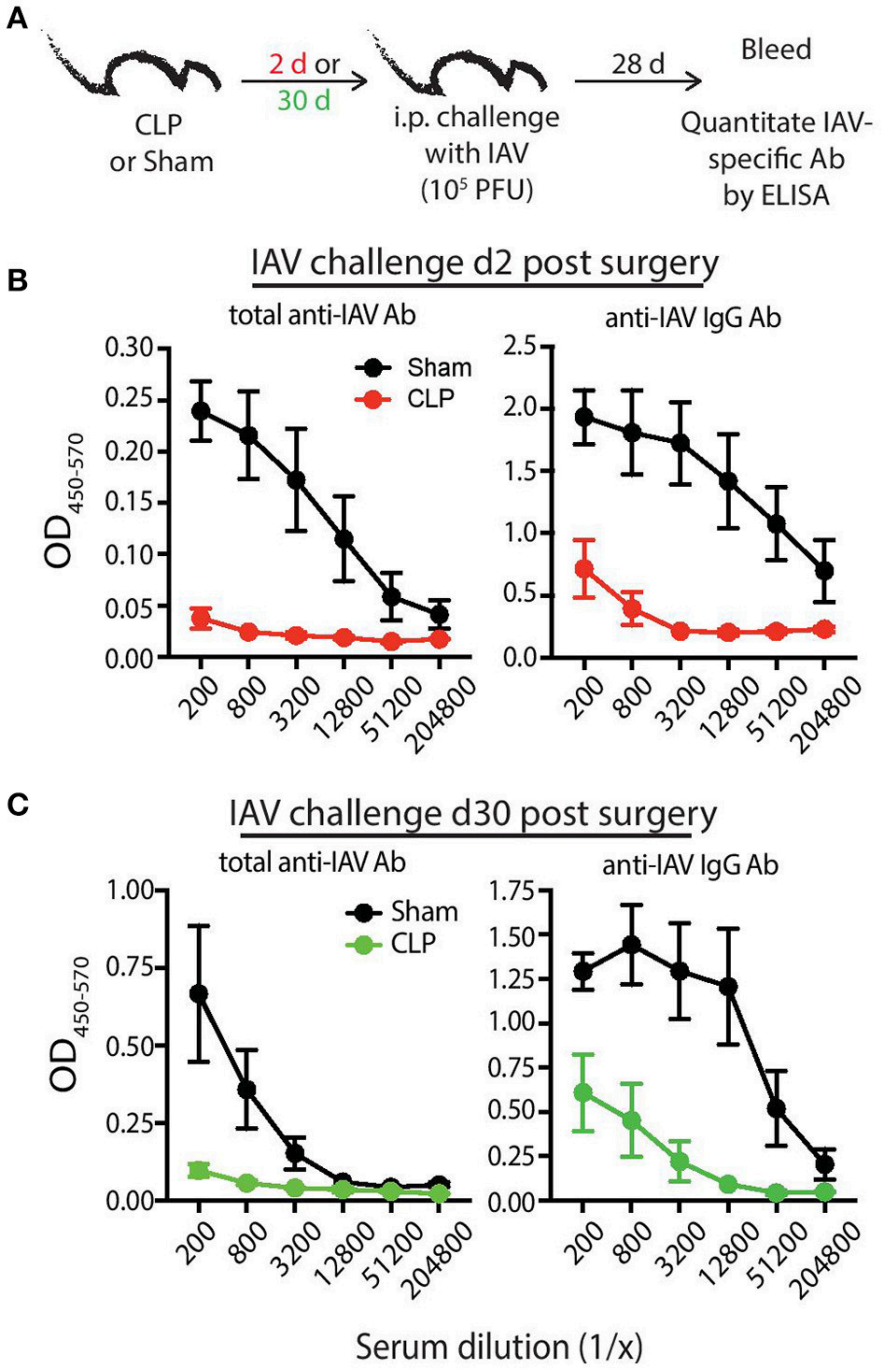
Septic mice have reduced primary Ab response following influenza A virus (IAV) challenge. **(A)** Experimental design—B6 mice were challenged with live IAV (A/PR/8; 10^5^ PFU in 100 μl PBS i.p.) 2 or 30 days after sham or CLP surgery. Serum was collected 28 days after immunization. **(B)** Absorbance of total anti-IAV Ab (left) and IgG specific for IAV (right) from the indicated dilutions of serum from mice challenged 2 days after surgery. **(C)** Absorbance of total anti-IAV Ab (left) and IgG specific for IAV (right) from the indicated dilutions of serum from mice challenged 30 days after surgery. *n* = 4–6 mice/group. Data are representative of at least 2 independent experimental replicates.

**Figure 7:**
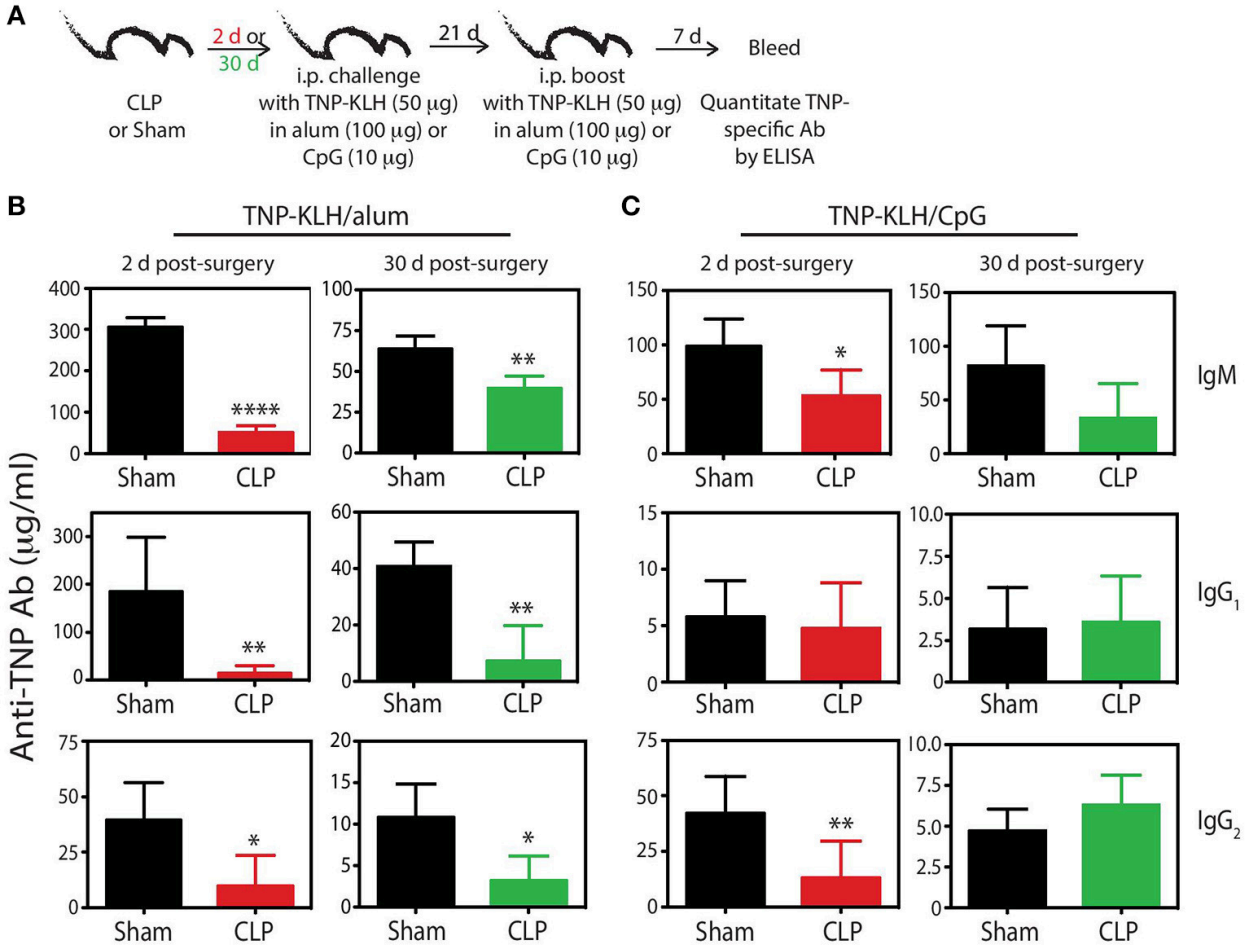
Generation of anti-TNP mAb is reduced in septic mice**. (A)** Experimental design—B6 mice were immunized i.p. with 50 μg TNP-KLH in alum or CpG 2 or 30 days after sham or CLP surgery. A second immunization was administered 21 days later. Blood was collected for serum 7 days after the second immunization. Serum concentrations (quantified via ELISA) of anti-TNP Ab from **(B)** TNP-KLH/alum- and **(C)** TNP-KLH/CpG-immunized mice 2 and 30 days after sham and CLP surgeries. *n* = 4–6 mice/group. *p < 0.05, **p < 0.01, ****p < 0.001. Data are representative of at least 2 independent experiments.

To bolster our findings above, we wanted to determine the impact of sepsis on Ab production during a primary CD4 T cell-dependent B cell response to a pathogen challenge commonly used to probe B cell and/or T cell response and a second model Ag classically used to evaluate the fitness of the humoral arm of adaptive immunity. B6 mice were challenged with live influenza A virus (IAV) on day 2 or 30 after sham or CLP surgery, and serum was collected 28 days later to measure the amount of anti-IAV Ab produced (Figure 6A). We noted marked reductions in the amount of total Ab and IgG specific for IAV in the serum of CLP-treated mice challenged on days 2 or 30 after CLP surgery (Figures 6B,C). Similar results were seen after immunization with TNP-KLH, a common Ag used to test various aspect of humoral immunity (Figure 7). Interestingly, the sepsis-induced deficiency Ab production was most pronounced and sustained when the TNP-KLH was administered with the Th2-polarizing adjuvant alum (49, 50) (Figure 7B). There remained a significant reduction in anti-TNP IgM, IgG_1_, and IgG_2_ even when the mice were first immunized 30 days after CLP surgery, and boosted 21 days later. By comparison, CLP-treated mice demonstrated a significant reduction in anti-TNP IgM and IgG_2_ when immunized with TNP-KLH mixed with Th1-polarizing adjuvant CpG (51) only on day 2, but these reductions were not maintained when immunization occurred 30 days after surgery (Figure 7C). The differences in response depending on the adjuvant used are intriguing, but it is important to note that our primary goal of these experiments was not to directly compare “Th1” vs. “Th2” priming conditions. Rather, we reasoned the comparison between control (sham) to CLP mice after the same duration post-surgery was more critical. Sham surgery likely causes some low-level abdominal inflammation, as the abdomen/peritoneum is surgically opened and the cecum exposed and put back into the mouse (without any needle puncture) followed by closure. While the mice received surgery at the same time, the peritoneum is going to be much different 2 days after this surgical event compared with 30 days where healing has taken place. Together, these results highlight the compromised ability to produce Ab during a primary B cell response following a septic event.

## Discussion

Defects in humoral immunity are associated with increased susceptibility to infection (52). Reduced immune function after septic injury is well-documented in the clinic, and a number of mechanisms have been posited to explain sepsis-induced immune suppression (12). Clinical data show acute reduction in both CD4 T cells and B cells in sepsis patients (20), as well as IgM levels in the circulation (53). The reduction of these components of the adaptive immune system contribute to the increased risk of nosocomial bacterial infections and viral reactivation, and poor chances for a favorable outcome (36, 54). While there is a reasonable understanding of the numerical changes in total B cells and CD4 T cells within several days after sepsis onset, there is a paucity of information describing the qualitative long-term impact of sepsis on these cells within the context of a primary CD4 T cell-dependent B cell response. The reduced Ab response following antigenic challenge in mice that experienced CLP-induced sepsis we have described here is consistent with the recent data by Mohr et al. (37). Further, the data we have presented importantly extend our understanding of what happens to the cellular components of the adaptive immune system following a septic event that affect the generation of a primary CD4 T cell-dependent B cells response. Our results show that following sepsis, mice subjected to CLP surgery have long-term reductions in B cell differentiation and class switching after vaccination or infection, ultimately resulting in suboptimal Ab production. The data reported here also suggest the reduced Ab production in mice challenged with Ag early during the septic event or late after sepsis resolution is due in part to insufficient help from Tfh cells, leading to inadequate B cell differentiation and class switching.

Work reported in a number of publications have examined basic numerical and functional changes of various immune cell subsets after sepsis (almost) exclusively at the total population level. In the present study we have evaluated endogenous Ag-specific CD4 T cell and B cell populations within the context of a CD4 T cell-dependent B cell response following a septic event. Tracking Ag-specific T cells and B cells permits the most rigorous and sensitive functional analysis of these cells during the response to vaccination or infection by allowing us to identify changes at the Ag-specific level that may not be resolvable when examining the total populations (23). Additionally, we were able to evaluate the sepsis-induced numerical and phenotypic changes in endogenous Ag-specific CD4 T cells and B cells (2W1S:I-A^b^- and PE-specific, respectively) responding to the same foreign Ag (2W1S peptide covalently coupled to PE in CFA) (28). As expected, the 2W1S:I-A^b^-specific CD4 T cells expand and differentiate into Tfh cells as well as other defined CD4 T cell subsets, such as Tbet^+^ “Th1” CD4 T cells (Figure 5D), in sham mice after 2W1S-PE/CFA vaccination. PE-specific B cells can then interact with the 2W1S:I-A^b^-specific Tfh cells within the GC. There is robust expansion, differentiation, and class switching seen in the PE-specific B cells in this setting. Our data suggest all of these factors are affected by sepsis, even once the host has recovered from the acute hyperinflammatory response and transient lymphopenia characteristic of a septic event.

While there were a number of results that were expected, our analyses also revealed some unexpected findings. For example, the total number of PE-specific B cells was not different in the sham and day 30 CLP groups (see Figure 2C). There was just a redistribution among the different subsets. CLP-induced polymicrobial sepsis induces a highly inflammatory event within the peritoneum, making it possible that this inflammation can drive a small number of the PE-specific cells to differentiate into GC B cells and plasma cells. The small but significant numerical increase in PE-specific GC B cells and plasma cells 30 days after CLP, prior to 2W1S-PE/CFA immunization (Figures 2E,F) was surprising, and was in contrast to the reduction seen in total 2W1S:I-A^b^-specific CD4 T cells (Figure 3C). While 2W1S:I-A^b^-specific CD4 T cells recognize a defined peptide sequence presented by MHC II, PE is a 250 kD multi-subunit protein originally isolated from red algae with multiple epitopes available for Ab recognition (55, 56). Thus, while being PE-specific, the B cells we detect using the enrichment protocol are likely polyclonal in composition, and bind to different epitopes within the PE protein. One possible explanation for the increase in PE-specific GC B cells and plasma cells 30 days after CLP is that some of the PE-specific B cells cross-react with antigenic epitopes expressed by the numerous gut commensal microbes released during the CLP surgery that establish the polymicrobial peritonitis. Such cross reactivity among B cells has been observed previously; for example, gut commensal bacteria can prime for the production of antibodies specific for HIV-1 envelope gp41 (57). Similarly, CD4 T cell cross-reactivity with gut commensal bacteria can drive responses to self Ag (glucose-6-phosphate isomerase) and foreign microbes that ultimately has an impact on host health (58–61). Future studies are needed to investigate this interesting possibility of cross-reactivity. Despite the increases seen in the PE-specific B cell populations after CLP, sepsis dramatically reduced the ability of these cells to respond to their cognate Ag following 2W1S-PE/CFA immunization. 2W1S:I-A^b^-specific CD4 T cells have reduced proliferative capacity and cytokine production after sepsis, as well has developing changes within the TCR Vβ repertoire (22). In addition, numerical and functional deficits occur among dendritic cells following sepsis (45), suggesting the potential contribution for both T cell-intrinsic and -extrinsic factors to reduced function.

Another interesting finding was the skewing of the Ab response in the mice subjected to CLP surgery to IgG2b after immunization (and even prior to immunization). The presence of TGF-β, which has been reported to be elevated after sepsis (62), promotes switching to IgG2b and IgA (63). While class switching to IgG2b was elevated across the board, IgA was slightly elevated only in GC B cells and plasma B cells in unimmunized mice. There is clear redundancy in regard to the ability of certain cytokines to drive IgG2 (as well as other) isotype switching in murine B cells. IFNγ and type I IFN can promote switching to IgG2, as exemplified by the presence of normal levels of induced IgG2b in mice unable to express the type II TGF-β receptor on their B cells (64). Interestingly, it has also been reported that the absence of T cell help and presence of LPS favors switching to IgG2b (63). Thus, given these redundancies and variety of cytokines produced during a septic event, we hesitate to suggest TGF-β (or any other single cytokine) is solely responsible for the IgG2b skewing after CLP.

While it is difficult to know how long the effects of a septic event will have on the function of the immune system, sepsis survivors have decreased 5-year survival compared to “control” patients (16, 65, 66). Perturbations, such as sepsis, that result in long-term impairments to the immune system have the potential to severely diminish vaccination efficacy. Typically, adults are recommended to receive a number of vaccinations to seasonal (e.g., influenza) and non-seasonal (e.g., pneumococcus and varicella zoster virus) pathogens to develop and maintain adequate protection from infection (67). These vaccinations work best when the host immune system is optimally functional. However, in a patient with a history of sepsis, these vaccinations may provide little-to-no protection, leaving these patients with an increased risk of secondary infection.

## Author contributions

FS, SC, JK, KP, CD, DD, TK, LT, KM, JC-P, and TG performed experiments and analyzed data. TW, VB, and TG provided input on the research design, and FS, SC, JK, CD, DD, TW, VB, TG wrote and edited the manuscript. All authors read and approved the submitted version.

### Conflict of interest statement

The authors declare that the research was conducted in the absence of any commercial or financial relationships that could be construed as a potential conflict of interest.

## Supplementary material

Supplemental Figure 1 Morbidity, cytokine production, and mortality following CLP surgery. B6 mice underwent sham or CLP surgery. **(A)** Body weight was measured before and the 7 days after surgery. Weight loss for each was determined based on their starting weight. **(B)** Serum samples were collected at the indicated time points after sham or CLP surgery. The amount of IL-1β, IL-6, IFNγ, and TNF in samples was determined by bioplex. *n* = 11 sham and 38 CLP mice for **(A,C)**; *n* = 5 mice/time point/group in **(B)** ^*^*p* < 0.05; ^**^*p* < 0.01, ^****^*p* < 0.001 for SPF—CLP vs. cohoused—CLP at the indicated time points.
