# Supplementary figures and images for: Polymicrobial Sepsis Chronic Immunoparalysis Is Defined by Diminished Ag-Specific T Cell-Dependent B Cell Responses
Source: Front Immunol. 2018 Oct 31;9:2532. doi: 10.3389/fimmu.2018.02532 (PMC6220049; doi:10.3389/fimmu.2018.02532)

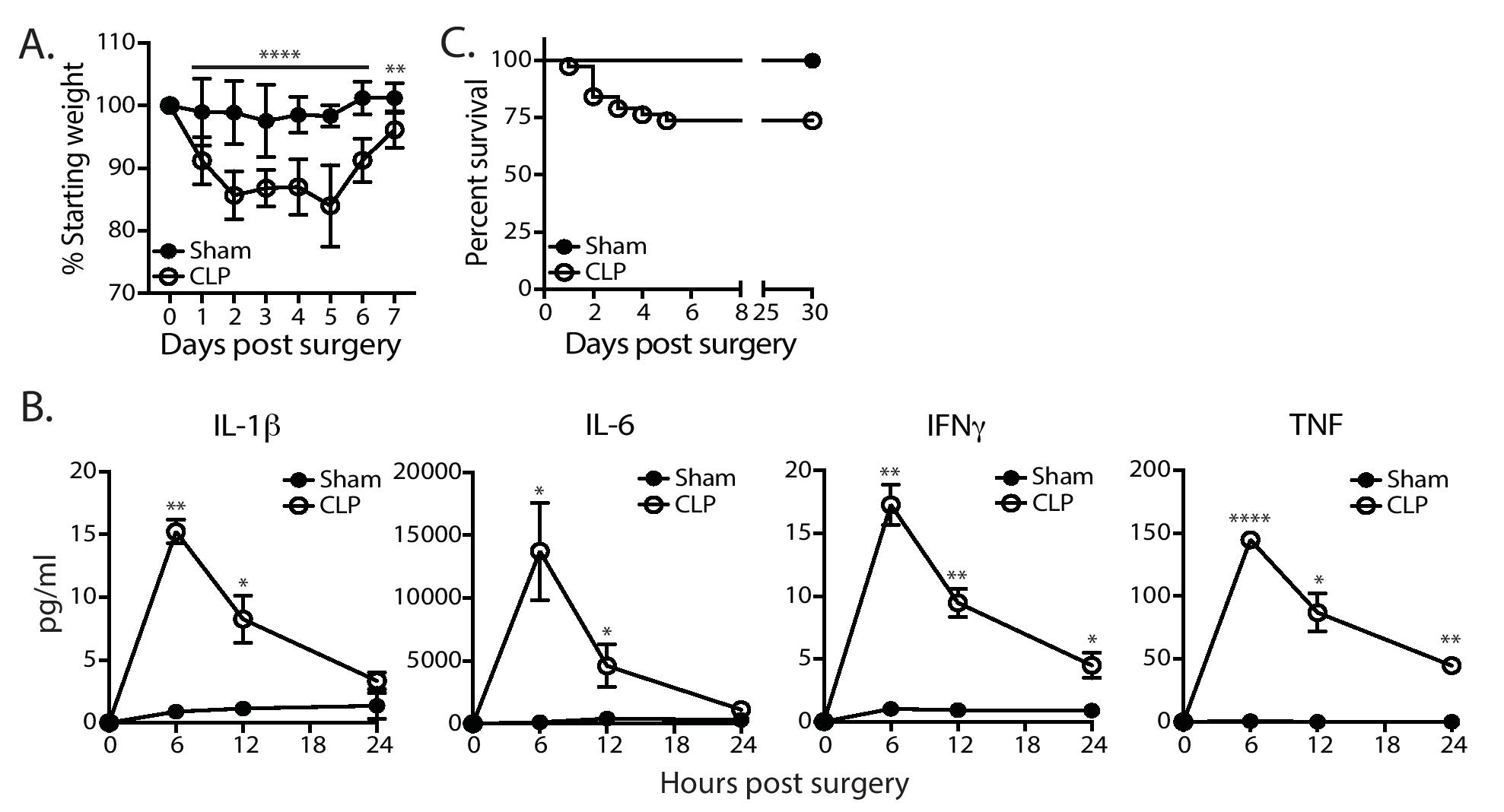

Supplement: Supplemental Figure 1 — Morbidity, cytokine production, and mortality following CLP surgery. B6 mice underwent sham or CLP surgery. (A) Body weight was measured before and the 7 days after surgery. Weight loss for each was determined based on their starting weight. (B) Serum samples were collected at the indicated time points after sham or CLP surgery. The amount of IL-1β, IL-6, IFNγ, and TNF in samples was determined by bioplex. n = 11 sham and 38 CLP mice for (A,C); n = 5 mice/time point/group in (B) *p < 0.05; **p < 0.01, ****p < 0.001 for SPF—CLP vs. cohoused—CLP at the indicated time points. [file Image_1.JPEG]
